# Liraglutide Activates mTORC1 Signaling and AMPA Receptors in Rat Hippocampal Neurons Under Toxic Conditions

**DOI:** 10.3389/fnins.2018.00756

**Published:** 2018-10-23

**Authors:** Sung Woo Park, Rodrigo B. Mansur, Yena Lee, Jae-Hon Lee, Mi Kyoung Seo, Ah Jeong Choi, Roger S. McIntyre, Jung Goo Lee

**Affiliations:** ^1^Paik Institute for Clinical Research, Inje University, Busan, South Korea; ^2^Department of Health Science and Technology, Graduate School, Inje University, Busan, South Korea; ^3^Department of Convergence Biomedical Science, College of Medicine, Inje University, Busan, South Korea; ^4^Mood Disorders Psychopharmacology Unit, University Health Network, University of Toronto, Toronto, ON, Canada; ^5^Department of Psychiatry, University of Toronto, Toronto, ON, Canada; ^6^Department of Psychiatry, National Rehabilitation Center, Seoul, South Korea; ^7^Department of Pharmacology, University of Toronto, Toronto, ON, Canada; ^8^Department of Psychiatry, College of Medicine, Haeundae Paik Hospital, Inje University, Busan, South Korea

**Keywords:** mammalian target of rapamycin complex 1 signaling, α-amino-3-hydroxy-5-methylisoxazole-4-propionic acid receptor, glucagon-like peptide 1 receptor, liraglutide, neuroplasticity, toxic condition

## Abstract

The aim of the present study was to determine whether treatment with liraglutide, a glucagon-like peptide 1 (GLP-1) receptor agonist, would alter mammalian target of rapamycin complex 1 (mTORC1) signaling and/or α-amino-3-hydroxy-5-methylisoxazole-4-propionic acid (AMPA) receptor activity under dexamethasone-induced toxic conditions. Western blot analyses were performed to assess changes in mTORC1-mediated proteins, brain-derived neurotrophic factor (BDNF), and various synaptic proteins (PSD-95, synapsin I, and GluA1) in rat hippocampal cultures under toxic conditions induced by dexamethasone, which causes hippocampal cell death. Hippocampal dendritic outgrowth and spine formation were measured using immunostaining procedures. Dexamethasone significantly decreased the phosphorylation levels of mTORC1 as well as its downstream proteins. However, treatment with liraglutide prevented these reductions and significantly increased BDNF expression. The increase in BDNF expression was completely blocked by rapamycin and 2,3-dioxo-6-nitro-1,2,3,4-tetrahydrobenzo[f]quinoxaline-7-sulfonamide (NBQX). Liraglutide also recovered dexamethasone-induced decreases in the total length of hippocampal dendrites and reductions in spine density in a concentration-dependent manner. However, the positive effects of liraglutide on neural plasticity were abolished by the blockade of mTORC1 signaling and AMPA receptors. Furthermore, liraglutide significantly increased the expression levels of PSD-95, synapsin I, and GluA1, whereas rapamycin and NBQX blocked these effects. The present study demonstrated that liraglutide activated mTORC1 signaling and AMPA receptor activity as well as increased dendritic outgrowth, spine density, and synaptic proteins under toxic conditions in rat primary hippocampal neurons. These findings suggest that GLP-1 receptor (GLP-1R) activation by liraglutide may affect neuroplasticity through mTORC1 and AMPA receptors.

## Introduction

Depressive disorder is a very common psychiatric illness that typically has a chronic course (Andrade et al., 2003). The lifetime prevalence of depressive disorder is approximately 17% in the United States (Andrade et al., 2003; Kessler et al., 2003). In addition to experiencing a depressed mood, patients with depressive disorder may exhibit compromised social functioning, decreased quality of life, and an elevated risk of medical illnesses (Brown et al., 2009; Dhar and Barton, 2016; Kupferberg et al., 2016). Thus, the burden of illness associated with depressive disorder necessitates genuinely novel treatment approaches (Millan et al., 2015). More than 50 years after the monoamine hypothesis of depression has been proposed, several monoamine-based antidepressants have been developed. It is now widely used as a pharmacological treatment for depression (Hillhouse and Porter, 2015). But, many difficulties are still associated with pharmacotherapies for depressive disorder (Lee et al., 2010; Popa-Velea et al., 2015). Thus, there is a need for innovative treatment strategies that utilize newly identified molecular mechanisms.

Novel agents that target molecular effector systems relevant to neuroplasticity and neurogenesis represent a promising research opportunity for psychiatry (Massart et al., 2012; Vialou et al., 2013; Pilar-Cuellar et al., 2014). In terms of neuroplastic mechanisms, much attention has been focused on plastic changes in neural connectivity that operate via the mammalian target of rapamycin complex 1 (mTORC1) pathway (Li et al., 2010; Duman et al., 2012). For example, Li et al. (2010) observed that the *N*-methyl-D-aspartate (NMDA) receptor antagonist ketamine decreases immobility time in the forced swimming test (FST), activates mTORC1 signaling, and increases synaptic protein levels (PSD-95, synapsin I, and GluA1) in the prefrontal cortex of mice. Furthermore, Maeng et al. (2008) demonstrated that the activation of mTORC1 signaling by ketamine and the associated antidepressant behavioral responses require the activation of α-amino-3-hydroxy-5-methylisoxazole-4-propionic acid (AMPA) receptors. The activations of AMPA and neurotrophic receptors play important roles in the synthesis of synaptic proteins that support synaptic plasticity (Ignacio et al., 2016).

Glucagon-like peptide 1 (GLP-1) is an endogenous incretin hormone composed of 30 amino acids that exerts differential effects based on its synthesis site and release (Holst et al., 2011). When GLP-1 is released in response to fat and carbohydrates by small intestinal L-cells, the activation of the GLP-1 receptor (GLP-1R) stimulates glucose-dependent insulin secretion, the synthesis of insulin, β-cell proliferation, and the inhibition of β-cell apoptosis (Drucker, 2001; Zhou et al., 2015). In contrast, when GLP-1 is released in the brain, it functions as a neuropeptide (Holst et al., 2011). GLP-1Rs are widely expressed throughout the brain in rodents and humans in areas such as the caudate putamen, cerebral cortex, and hippocampus (Perry et al., 2002; Perry and Greig, 2004; Li M. et al., 2015). Accumulating evidence indicates that GLP-1 acts as a neuronal growth factor and it has been shown that GLP-1 enhances neurite outgrowth and protects against oxidative injury in PC12 and SK-N-SH human neuroblastoma cells (Li M. et al., 2015).

Liraglutide, which is a GLP-1R agonist, is approved by the U.S. Food and Drug Administration as an adjunct to diet and exercise and is aimed at improving weight loss and glycemic control in adults with type 2 diabetes (Gross, 2013). However, in addition to its anti-diabetic treatment effects, recent studies have shown that liraglutide may have neuroprotective effects and can modulate neuroplasticity as well as cognitive function. Liraglutide exerts neurotrophin-like activity at least in part via the MEK-ERK signaling pathway (Li M. et al., 2015) and its neuroprotective effects appear to operate via reductions in excessive levels of reactive oxygen species (ROS; Zhu et al., 2016). Liraglutide also decreases the hyperphosphorylation of tau and neurofilament proteins and improves learning and memory ability in mice (Xiong et al., 2013). Although several studies have suggested possible molecular mechanisms underlying the effects of liraglutide (O’Neill, 2013), no studies have investigated the neuroplastic effects of liraglutide and its association with mTORC1 signaling or AMPA receptors. Thus, the present study aimed to determine whether there is an association of the neuroplastic effects of liraglutide with the activations of mTORC1 signaling and AMPA receptors. For this study, we used a synthetic glucocorticoid dexamethasone as neurotoxic model for screening the neuroplastic effects of liraglutide. Excessive glucocorticoid levels in depression lead to hippocampal atrophy and disturbances of neuroplasticity (McEwen et al., 1992). Dexamethasone in neuronal cells was reported to reduce cell viability, cell proliferation, and neurite outgrowth (Obradovic et al., 2006; Leskiewicz et al., 2013). Thus, the effects of liraglutide on mTORC1 signaling, AMPA receptor activity, synaptic protein expression, neurite outgrowth, and synaptic density were investigated using rat primary hippocampal neurons under dexamethasone-induced toxic conditions.

## Materials and Methods

### Drugs and Reagents

Liraglutide and dexamethasone were purchased from GL Biochem Ltd. (Shanghai, China) and Sigma (St. Louis, MO, United States), respectively. The reagents used for the primary hippocampal cultures were purchased from Invitrogen (Carlsbad, CA, United States) and included dimethyl sulfoxide (DMSO), neurobasal medium, fetal bovine serum (FBS), horse serum (HS), B27 supplement, L-glutamine, penicillin-streptomycin, and trypsin. The antibodies used for the Western blot analyses were purchased from the following sources: anti-BDNF (sc-546), anti-synapsin I (sc-8295), anti-rabbit (sc-2004), and anti-goat (sc-2020) IgG-horseradish peroxide-conjugates from Santa Cruz Biotechnology (Santa Cruz, CA, United States); monoclonal anti-α-tubulin (T9026) and anti-mouse IgG peroxidase conjugates (A4416) from Sigma; anti-phospho-mTORC1 (Ser2448, #2971), anti-mTORC1 (#2972), anti-phospho-4E-BP-1 (Thr37/46, #2855), anti-4E-BP-1 (#9452), anti-phospho-p70S6K (Thr389, #9205), and anti-p70S6K (#9202) from Cell Signaling Technology (Beverly, MA, United States); anti-PSD-95 (AB9634) from Millipore (Temecula, CA, United States); and anti-GluA1 (ab109450) from Abcam (Cambridge, United Kingdom).

The antibodies used for immunostaining were purchased from the following sources: anti-microtubule-associated protein 2 (MAP2; MAB3418) from Millipore; Alexa Fluor^®^ 568 goat anti-mouse IgG (A11031) and Hoechst 33258 (H21491) from Invitrogen; and Alexa Fluor^®^ 488 Phalloidin from Molecular Probes (Eugene, OR, United States). Specific kinase inhibitors were purchased from the following sources: PI3K inhibitor LY294002 from Cell Signaling Technology; MEK inhibitor PD98059 and mTORC1 inhibitor rapamycin from Calbiochem (San Diego, CA, United States); and the AMPA receptor inhibitor 2,3-dioxo-6-nitro-1,2,3,4-tetrahydrobenzo[f] quinoxaline-7-sulfonamide (NBQX) from Tocris Bioscience (Ballwin, MO, United States).

### Primary Hippocampal Cell Cultures

Pregnant Sprague-Dawley rats were obtained from Orient Bio (Seongnam, South Korea) and all experimental procedures were approved by the Inje Medical College Committee for Animal Experimentation and the Institutional Animal Laboratory Review Board (Approval no. 2015-027).

Hippocampal cell cultures were prepared from the hippocampi of fetuses at embryonic day 17 in a manner similar to that reported by Kaech and Banker (2006). Briefly, the hippocampi were carefully removed and dispersed in neurobasal medium containing 0.03% trypsin for digestion at 37°C (5% CO2) for 20 min. Next, the cells were suspended in a medium including 1% FBS, 1% HS, 2% serum-free growth medium B27, 0.25% L-glutamine, and 50 U/ml penicillin-streptomycin; this was the control condition. For the Western blot analyses, the cells were plated on 6-well dishes coated with poly-L-lysine at a density of 2 × 10^5^ cells per dish. For the neurite and spine assays, the cells were plated on 12-well dishes at a density of 2 × 10^4^ or 5 × 10^3^ cells per dish. They were grown under the control condition for either 7 days (for the neurite and spine assays) or 10 days (for Western blots). After incubation for 7 or 10 days, the cells were treated with liraglutide in the presence or absence of 500 μM dexamethasone for either 4 days (for the Western blots) or 5 days (for the neurite and spine density assays) before being harvested for further analysis. In a preliminary experiment performed by our research group, exposure to 500 μM dexamethasone caused a significant reduction in hippocampal cell viability (approximately 22% decrease; Supplementary Figure 1).

### Drug Treatment

Liraglutide (1 μM) was completely dissolved in distilled water and then diluted to various concentrations also using distilled water prior to use. Dexamethasone (50 mM) was dissolved in DMSO (Amresco, OH, United States) and then diluted to a final concentration of 500 μM using 1% DMSO prior to use. For the Western blot analyses, the cells were cultured for 4 days with liraglutide (10 or 100 nM) and, for the neurite and spine density assays, the cells were cultured for 5 days with liraglutide (100 nM) in the presence or absence of dexamethasone. The range of liraglutide concentration used in the present study was based on studies showing that liraglutide exerts neuroprotective effects in various cell cultures (Miao et al., 2013; Sharma et al., 2014; Li Y. et al., 2015).

### Western Blot Analysis

The Western blot experiments were performed as previously described (Seo et al., 2014). Briefly, the hippocampal cells were homogenized in ice-cold lysis buffer (20 mM Tris–HCl, 137 mM NaCl, 10% glycerol, 1% Nonidet^TM^ P-40, 0.1% sodium dodecyl sulfate [SDS], 0.5% sodium deoxycholate, and 2 mM ethylenediaminetetraacetic acid [EDTA]) and one complete protease inhibitor tablet (Roche; Laval, QC, Canada). The lysates were centrifuged (1000 × g, 15 min, 4°C) and the samples were stored at -80°C until use. Immunoblotting was performed with one of the primary antibodies (anti-phospho-mTORC1, anti-mTORC1, anti-phospho-4E-BP-1, anti-4E-BP-1, anti-phospho-p70S6K, anti-p70S6K, anti-BDNF, anti-PSD-95, anti-synapsin I, or anti-GluA1 [1:1000] and anti-α-tubulin [1:2000]) in Tris-buffered saline with Tween-20 (TBS-T) at 4°C overnight and then the membranes were washed three times in TBS-T for 10 min. The membranes were then incubated for 1 hr in TBS-T containing a horseradish peroxidase-conjugated secondary antibody (goat-anti-rabbit IgG for anti-phospho-mTORC1, anti-mTORC1, anti-phospho-4E-BP-1, anti-4E-BP-1, anti-phospho-p70S6K, anti-BDNF, anti-p70S6K, anti-phospho-eIF4B, anti-eIF4B, anti-phospho-S6, anti-S6, anti-BDNF, anti-PSD-95, or anti-GluA1 [1:2000]; donkey-anti-goat IgG for anti-synapsin I [1:2000]; or anti-mouse IgG for anti-α-tubulin [1:10,000]) at room temperature. The Western blot analyses were repeated two times per group for each of the two independent cultures.

### Neurite Assay

Dendrites were visualized via immunostaining using a MAP-2 antibody, which is a dendritic marker, as previously described (Park et al., 2016). To analyze total dendritic length, five fields were randomly selected from each group and two independent cultures were performed. All neurons in a given field were counted, including both basal and apical dendrites, and dendritic length was determined to be the distance between the edge of the cell body and the tip of the growth cone. Total dendritic length was obtained by summing the lengths of all dendrites from a single neuron and then averaging this measure in each group using MetaMorph (Molecular Devices, Downingtown, PA, United States), an automated image-analysis program (Klimaschewski et al., 2002). At least 400 cells were analyzed in 10 fields by a researcher blind to the groups.

### Spine Density Assay

Spines were stained with phalloidin as previously described (Park et al., 2016). To analyze spine density, spines, and filopodia were differentiated by shape and length such that spines were defined as less than 3 μm long with a rounder or mushroom shape while filopodia were defined as between 3 and 10 μm long with a narrower shape. Ten neurons were randomly selected from each group and two independent cultures were performed. Using 12–20 neurons per group, two dendritic segments per neuron (50 μm) were analyzed (23–40 dendritic segments per group) by a researcher blind to the groups. To represent average spine density in a 10-μm dendrite, the spine density of a 50-μm dendritic segment was divided by 5.

### Statistical Analysis

All statistical analyses were performed using GraphPad Prism software (ver. 7.03; GraphPad Software, La Jolla, CA, United States). Changes in the levels of mTORC1-mediated proteins were analyzed by one-way analysis of variance (ANOVA) followed by *post hoc* Tukey’s multiple comparisons. Two-way ANOVA was used to assess the main effect of liraglutide or the inhibitor and the interaction between liraglutide and the inhibitor; when warranted, Tukey’s multiple comparisons were carried out. *P*-values <0.05 were considered to indicate statistical significance.

## Results

### Effects of Liraglutide on mTORC1, 4E-BP-1, and p70SK Levels in Rat Hippocampal Cells Under the Dexamethasone-Induced Toxic Condition

**FIGURE 1:**
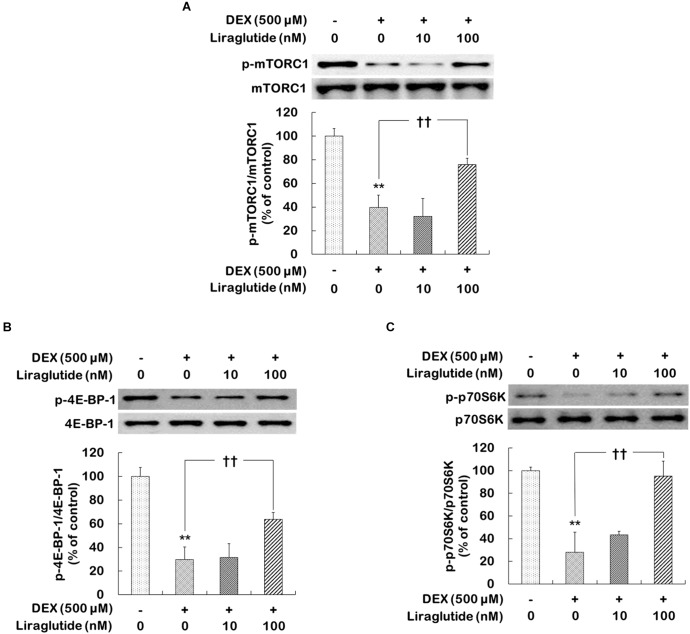
Effects of liraglutide on mTORC1, 4E-BP-1, and p70SK phosphorylation levels in hippocampal cells treated with dexamethasone. Cells were treated with liraglutide (10 or 100 nM) or distilled water (control) for 4 days either with (+; dexamethasone condition) or without (–; control condition) dexamethasone (DEX; 500 μM). In two different wells per group of each of two independent cultures (total 4 wells), cell lysates were analyzed by sodium dodecyl sulfate polyacrylamide gel electrophoresis (SDS-PAGE) and Western blot analyses with each of the primary antibodies. The Western blot analyses revealed the levels of phospho-Ser^2448^-mTORC1 **(A)**, phospho-Thr^37/46^-4E-BP-1 **(B)**, and phospho-Thr^389^-p70S6K **(C)**. Representative images and quantitative analyses normalized to the total levels for each protein are shown; values (*n* = 4) are shown as the mean ± standard error of the mean (SEM) expressed as a percentage of the control cell values. ^∗∗^*p* < 0.01 vs. control cells (-DEX, no liraglutide), ^††^*p* < 0.01 vs. dexamethasone-treated cells (+DEX, no liraglutide).

Dexamethasone treatment significantly reduced the phosphorylation levels of mTORC1, 4E-BP-1, and p70S6K (phospho-Ser^2448^-mTORC1 levels: 40% of control, *p* < 0.001; phospho-Thr^37/46^-4E-BP-1 levels: 30% of control, *p* < 0.001; phospho-Thr^389^-p70S6K levels: 28% of control, *p* < 0.001, Figures 1A–C). In the dexamethasone condition, liraglutide (100 nM) significantly prevented reductions in the phosphorylation levels of mTORC1, 4E-BP-1, and p70S6K (phospho-Ser^2448^-mTORC1 levels: ANOVA, *F*_[3,12]_ = 42.310, *p* < 0.001; *post hoc*, *p* = 0.001, Figure 1A; phospho-Thr^37/46^-4E-BP-1 levels: ANOVA, *F*_[3,12]_ = 49.800, *p* < 0.001; *post hoc*, *p* = 0.001, Figure 1B; phospho-Thr^389^-p70SK levels: ANOVA, *F*_[3,12]_ = 26.630, *p* < 0.001; *post hoc*, *p* < 0.001, Figure 1C). In addition, liraglutide prevented the dexamethasone-induced decrease in the phosphorylation levels of eIF4B and S6 (Supplementary Figures 2A,B).

### Activations of mTORC1 Signaling and AMPA Receptors Were Necessary for the Effects of Liraglutide on BDNF Expression in Rat Hippocampal Cells Under Dexamethasone-Induced Toxic Conditions

**FIGURE 2:**
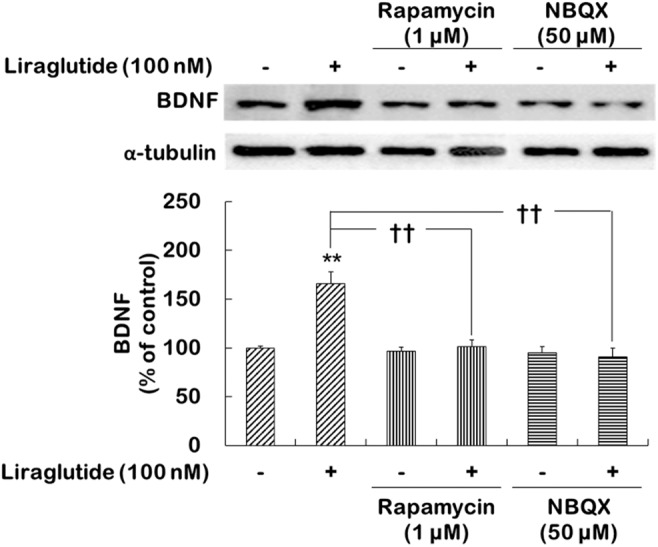
Effects of rapamycin and NBQX on the regulation of liraglutide-induced BDNF expression in hippocampal cells treated with dexamethasone. Cells were exposed to rapamycin (1 μM) or NBQX (50 μM) for 30 min prior to the addition of distilled water (control) or liraglutide (100 nM) for 4 days with dexamethasone (500 μM). In two different wells per group of each of two independent cultures (total 4 wells), cell lysates were analyzed by sodium dodecyl sulfate polyacrylamide gel electrophoresis (SDS-PAGE) and Western blot analyses for each of the primary antibodies. The Western blot analyses revealed the levels of BDNF. A representative image and quantitative analysis normalized to the α-tubulin band are shown. Values (*n* = 4) are mean ± standard error of the mean (SEM) expressed as a percentage of the control cell values. ^∗∗^*p* < 0.01 vs. control cells (no liraglutide and no inhibitors), ^††^*p* < 0.01 vs. liraglutide-only-treated cells.

The present study further examined whether the activations of mTORC1 signaling and AMPA receptors would contribute to the regulation of BDNF expression induced by liraglutide following the administration of dexamethasone. Two-way ANOVA revealed significant effects of liraglutide (*F*_[1,12]_ = 10.130, *p* = 0.008), rapamycin (*F*_[1,12]_ = 11.080, *p* = 0.006), and the liraglutide × rapamycin interaction (*F*_[1,12]_ = 8.279, *p* = 0.010) as well as liraglutide (*F*_[1,12]_ = 13.100, *p* = 0.004), NBQX (*F*_[1,12]_ = 7.774, *p* = 0.020), and the liraglutide × NBQX interaction (*F*_[1,12]_ = 10.140, *p* = 0.008) for BDNF expression. *Post hoc* analyses revealed that liraglutide significantly increased BDNF expression (*p* = 0.001) and that this increase was blocked by rapamycin (*p* = 0.002) and NBQX (*p* < 0.010; Figure 2).

### Effects of Liraglutide on Dendritic Outgrowth and Spine Formation in Rat Hippocampal Cells Under Dexamethasone-Induced Toxic Conditions

**FIGURE 3:**
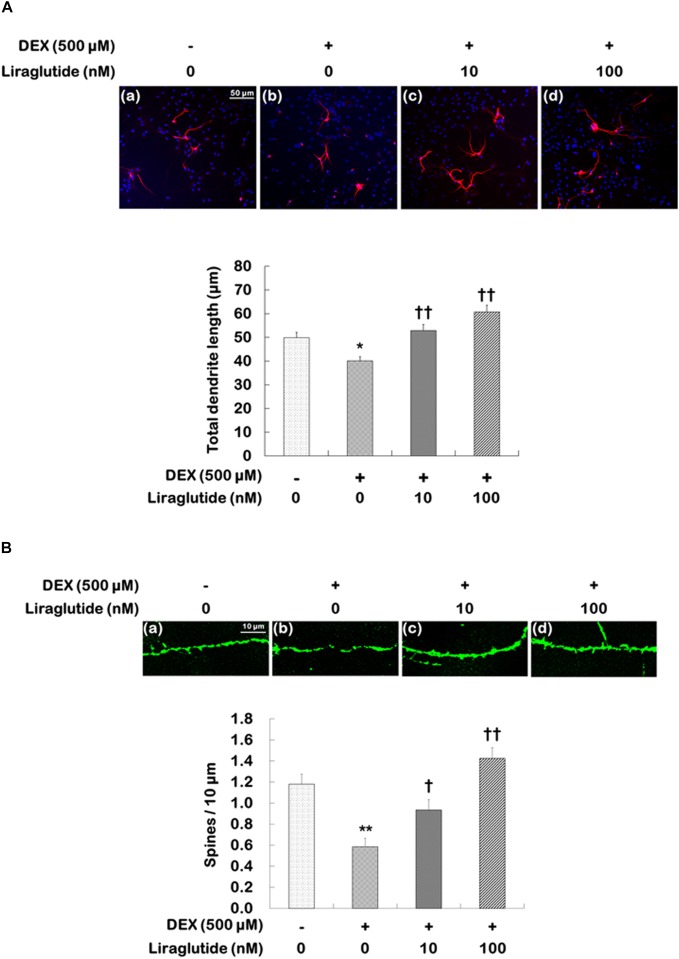
Effects of liraglutide on total dendritic length and spine density in hippocampal cells treated with dexamethasone. Cells were treated with liraglutide (10 or 100 nM) or distilled water (control) for 5 days either with (+; dexamethasone condition) or without (–; control condition) dexamethasone (DEX; 500 μM). Two independent cultures were performed and the cells were photographed and scored according to the methods described above. In total, 400 cells of group were analyzed for total dendritic length **(A)**. In total, 40 dendritic segments per group were analyzed for spine density **(B)**. All data (*n* = 400 for dendritic length, *n* = 40 for spine density) are expressed as mean ± standard error of the mean (SEM). ^∗^*p* < 0.05 vs. control cells (-DEX, no liraglutide), ^∗∗^*p* < 0.01 vs. control cells, ^†^*p* < 0.05 vs. dexamethasone-treated cells (+DEX, no liraglutide), ^††^*p* < 0.01 vs. dexamethasone-treated cells.

Hippocampal cells were incubated with liraglutide (10 or 100 nM) under either control or dexamethasone (500 μM) condition for 5 days. They were then photographed and assessed to quantify total dendritic length (Figure 3A) and spine density (Figure 3B) following treatment with liraglutide. Liraglutide did not affect total dendritic length or spine density in hippocampal cells under the control condition (Supplementary Table 1). In the absence of dexamethasone, control cells exhibited modest dendritic differentiation and an average dendritic length of approximately 50 μm (Figure 3A). On the other hand, dexamethasone-treated cells exhibited a decreased total dendritic length compared to control cells (dexamethasone-treated cells vs. control cells: 40 μm vs. 50 μm, *p* = 0.020, Figure 3A) but this reduction was prevented by liraglutide in a concentration-dependent manner (ANOVA; *F*[_3,1596]_ = 12.860, *p* < 0.001; 10 nM = 53 μm, *p* < 0.001; 100 nM = 61 μm, *p* < 0.001, Figure 3A).

The changes in spine density were similar to those observed for total dendritic length. Spine density was significantly reduced in dexamethasone-treated cells compared to that in control cells (dexamethasone-treated cells vs. control cells: 0.6 vs. 1.2, *p* < 0.001, Figure 3B), but liraglutide significantly prevented the reduction in dendritic spine density in a concentration-dependent manner under the dexamethasone condition (ANOVA; *F*_[3,156]_ = 17.000, *p* < 0.001; 10 nM = 0.9, *p* = 0.030; 100 nM = 1.4, *p* < 0.001, Figure 3B).

### Effects of mTORC1 and AMPA Receptor Inhibitors on Liraglutide-Induced Increases in Dendritic Outgrowth and Spine Formation

**FIGURE 4:**
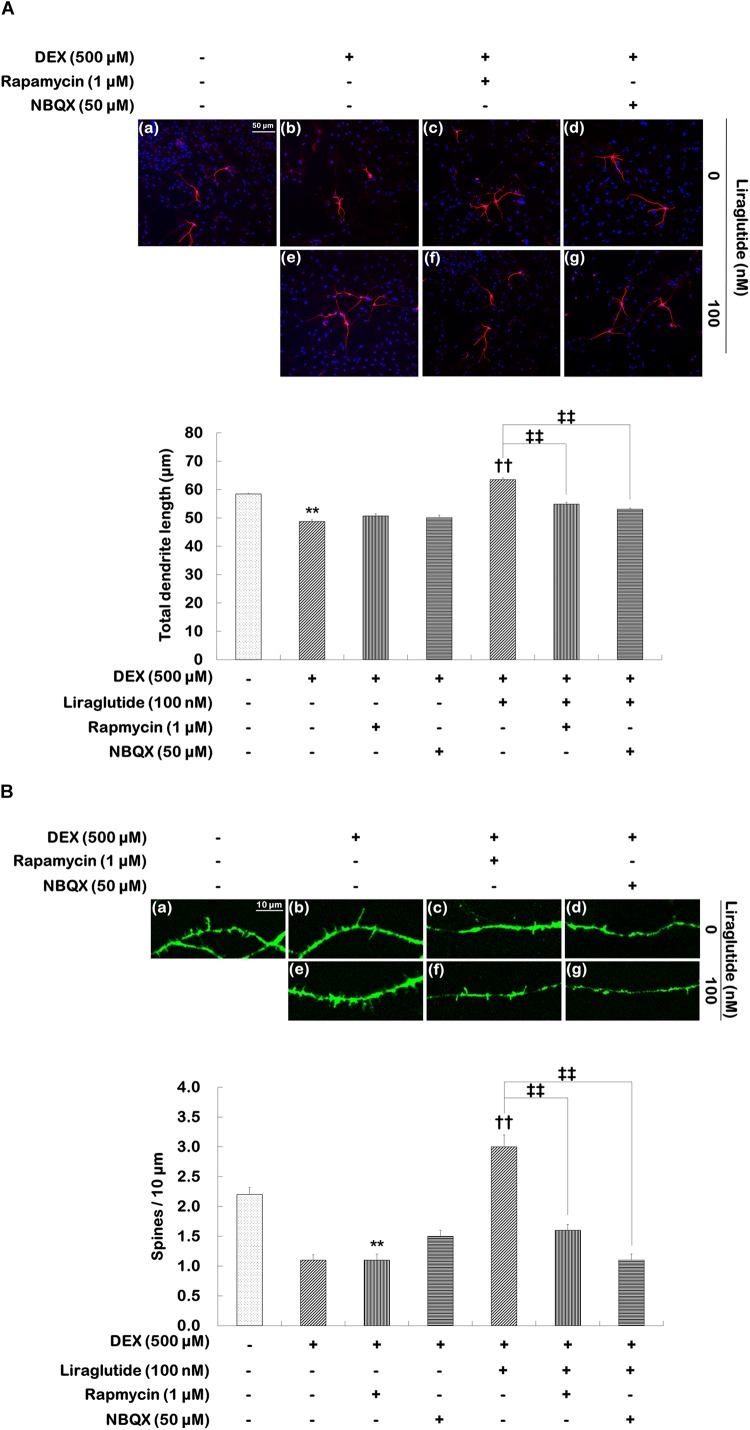
Effects of rapamycin and NBQX on liraglutide-induced increases in total dendritic length and spine density in hippocampal cells treated with dexamethasone. Cells were exposed to rapamycin (1 μM) and NBQX (50 μM) for 30 min prior to the addition of distilled water (control) or liraglutide (100 nM) for 5 days either with (+; dexamethasone condition) or without (–; control condition) dexamethasone (DEX; 500 μM). Two independent cultures were performed and cells were photographed and scored according to the methods described above. In total, 400 cells per group were analyzed for total dendritic length **(A)**. In total, 23–31 dendritic segments per group were analyzed for spine density **(B)**. All data (*n* = 400 for dendritic length, *n* = 23–31 for spine density) are expressed as mean ± standard error of the mean (SEM). ^∗∗^*p* < 0.01 vs. control cells (-DEX, no liraglutide and no inhibitors), ^††^*p* < 0.01 vs. dexamethasone-treated cells (+DEX, no liraglutide and no inhibitors), ^‡‡^*p* < 0.01 vs. dexamethasone and liraglutide-treated cells (+DEX, liraglutide only).

To investigate the roles that mTORC1 signaling and AMPA receptors play in the enhancement of liraglutide-induced dendritic outgrowth and spine formation, hippocampal cells were pretreated with rapamycin or NBQX under the dexamethasone condition. Two-way ANOVA revealed significant differences for rapamycin (*F*_[1,1596]_ = 23.380, *p* < 0.001), liraglutide (*F*_[1,1596]_ = 183.800, *p* < 0.001), and the rapamycin × liraglutide interaction (*F*_[1,1596]_ = 57.790, *p* < 0.001) as well as NBQX (*F*_[1,1596]_ = 50.610, *p* < 0.001), liraglutide (*F*_[1,1596]_ = 192.300, *p* < 0.001), and the NBQX × liraglutide interaction (*F*_[1,1596]_ = 86.210, *p* < 0.001) for total dendritic length. More specifically, rapamycin and NBQX inhibited the enhancement of total dendritic length induced by liraglutide (liraglutide vs. rapamycin + liraglutide: 64 μm vs. 55 μm, *p* < 0.001; liraglutide vs. NBQX + liraglutide: 64 μm vs. 53 μm, *p* < 0.001; Figure 4A).

Statistical analyses of spine density revealed significant effects of rapamycin (*F*_[1,101]_ = 29.840, *p* < 0.001), liraglutide (*F*_[1,101]_ = 78.260, *p* < 0.001), and the rapamycin × liraglutide interaction (*F*_[1,101]_ = 26.680, *p* < 0.001) as well as NBQX (*F*_[1,103]_ = 33.470, *p* < 0.001), liraglutide (*F*_[1,103]_ = 34.370, *p* < 0.001), and the NBQX × liraglutide interaction (*F*_[1,103]_ = 75.040, *p* < 0.001). *Post hoc* analyses showed that rapamycin and NBQX inhibited the liraglutide-induced enhancement of spine density (liraglutide vs. rapamycin + liraglutide: 3.0 vs. 1.6, *p* < 0.001; liraglutide vs. NBQX + liraglutide: 3.0 vs. 1.1, *p* < 0.001, Figure 4B).

### Activations of mTORC1 Signaling and AMPA Receptors Necessary for the Effects of Liraglutide on Synaptic Protein Expression in Rat Hippocampal Cells Under the Dexamethasone-Induced Toxic Condition

Whether liraglutide would similarly enhance the expression of various synaptic proteins, including PSD-95, synapsin I, and GluA1, through the activation of mTORC1 signaling and AMPA receptors was investigated in hippocampal cells under the dexamethasone condition.

**FIGURE 5:**
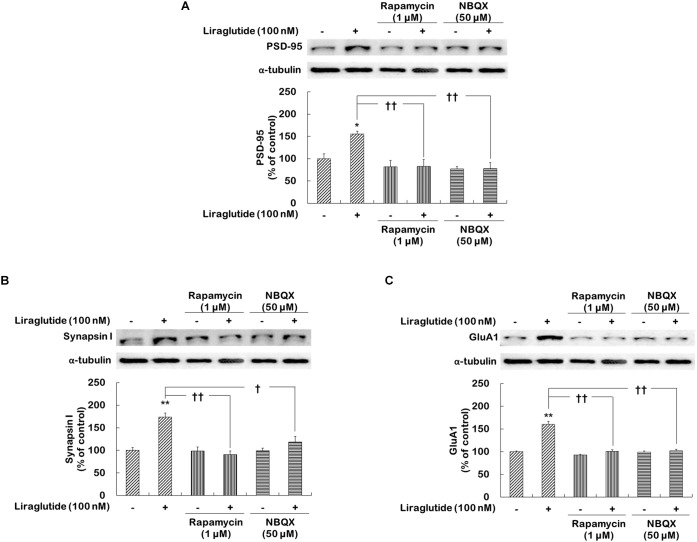
Effects of rapamycin and NBQX on the regulation of liraglutide-induced synaptic protein expression in hippocampal cells treated with dexamethasone. Cells were exposed to rapamycin (1 μM) or NBQX (50 μM) for 30 min prior to the addition of distilled water (control) or liraglutide (100 nM) for 4 days with dexamethasone (500 μM). In two different wells per group of each of two independent cultures (total 4 wells), cell lysates were analyzed by sodium dodecyl sulfate polyacrylamide gel electrophoresis (SDS-PAGE) and Western blot analyses for each of the primary antibodies. The Western blot and revealed the levels of PSD-95 **(A)**, synapsin I **(B)**, and GluA1 **(C)**. Representative images and quantitative analyses normalized to the α-tubulin band are shown. Values (*n* = 4) are mean ± standard error of the mean (SEM) expressed as a percentage of the control cell values. ^∗^*p* < 0.05 vs. control cells (no liraglutide and no inhibitors), ^∗∗^*p* < 0.01 vs. control cells, ^†^*p* < 0.05 vs. liraglutide-only-treated cells, ^††^*p* < 0.01 vs. liraglutide-only-treated cells.

Two-way ANOVA revealed significant effects of liraglutide (*F*_[1,12]_ = 26.750, *p* < 0.001), rapamycin (*F*_[1,12]_ = 10.370, *p* = 0.007), and their interaction (*F*_[1,12]_ = 9.630, *p* = 0.009) as well as main effects of liraglutide (*F*_[1,12]_ = 17.330, *p* = 0.001), NBQX (*F*_[1,12]_ = 5.381, *p* = 0.040), and their interaction (*F*_[1,12]_ = 5.186, *p* = 0.040) for PSD-95 expression; significant effects of liraglutide (*F*_[1,12]_ = 16.750, *p* = 0.008), rapamycin (*F*_[1,12]_ = 10.290, *p* = 0.001), and their interaction (*F*_[1,12]_ = 15.810, *p* = 0.002) as well as significant effects of liraglutide (*F*_[1,12]_ = 5.710, *p* = 0.030), NBQX (*F*_[1,12]_ = 15.630, *p* = 0.002), and their interaction (*F*_[1,12]_ = 5.349, *p* = 0.040) for synapsin I expression; and significant effects of liraglutide (*F*_[1,12]_ = 35.800, *p* < 0.001), rapamycin (*F*_[1,12]_ = 35.800, *p* < 0.001), and their interaction (*F*_[1,12]_ = 22.310, *p* < 0.001), as well as significant effects of liraglutide (*F*_[1,12]_ = 26.350, *p* < 0.001), NBQX (*F*_[1,12]_ = 31.440, *p* < 0.001), and their interaction (*F*_[1,12]_ = 26.350, *p* < 0.001) for GluA1 expression. Liraglutide significantly increased the expression of PSD-95 (156% of control, *p* = 0.020; Figure 5A), synapsin I (174% of control, *p* = 0.001; Figure 5B), and GluA1 (160% of control, *p* < 0.001; Figure 5C). However, the positive effects of liraglutide were blocked by rapamycin (*p* = 0.002 for PSD-95, Figure 5A; *p* < 0.001 for synapsin I, Figure 5B; and *p* < 0.001 for GluA1, Figure 5C) and NBQX (*p* = 0.001 for PSD-95, Figure 5A; *p* = 0.020 for synapsin I, Figure 5B; and *p* < 0.001 for GluA1, Figure 5C).

## Discussion

The present study demonstrated that liraglutide influenced mTORC1 signaling and AMPA receptor activities and was associated with changes in the expression of synaptic proteins, neurite outgrowth, and synaptic density in rat primary hippocampal neurons treated with toxic levels of dexamethasone.

Liraglutide is a long-acting human GLP-1 analog that has a 97% amino acid sequence identity with human GLP-1 (Miao et al., 2013). As a result, liraglutide is a potent antidiabetic agent that is protective against pancreatic β-cell apoptosis *in vitro* and increases pancreatic β-cell mass *in vivo* (Rolin et al., 2002; Bregenholt et al., 2005). In addition to these reported effects, liraglutide may also be neuroprotective and alter neuroplasticity in the brain (Holst et al., 2011; Hunter and Holscher, 2012; Xiong et al., 2013; Zhu et al., 2016). In preclinical studies, GLP-1 and its longer-lasting analogs decrease apoptosis, protect neurons from oxidative stress and inflammation, induce neurite outgrowth, protect neural plasticity, and enhance memory formation in the brains of animals in mouse models of Alzheimer’s disease, Parkinson’s disease, and other neurodegenerative diseases (Holscher, 2010, 2012; Xiong et al., 2013; Liu et al., 2015). In a study of GLP-1R knockout mice, GLP-1R-deficient mice exhibited a phenotype characterized by learning deficits that were restored by hippocampal Glp1r transfer (During et al., 2003). Additionally, GLP-1R affects neurite outgrowth and neuroplasticity. In a separate study, Li M. et al. (2015) found that liraglutide induces neurite outgrowth in C57BL/6 mouse primary cortical neurons and that this effect is blocked by the MEK-ERK inhibitor U0126. Hunter and Holscher (2012) reported that peripherally administered liraglutide and lixisenatide cross the blood–brain barrier in C57BL/6 mice where they enhance cyclic adenosine monophosphate (cAMP) levels and increase neurogenesis. Taken together, these results provide the basis for speculations that liraglutide may have neuroplastic effects.

However, the underlying mechanisms by which GLP-1 and GLP-1 analogs induce neuroplastic effects via mTORC1 signaling remained unclear. Most studies have examined the effects of GLP-1 or GLP-1 analogs on mTORC1 signaling and its protective effects using pancreatic β-cells. Miao et al. (2013) found that liraglutide increases INS-1 cells and activates mTOR as well as its downstream effectors, including 70-kDa ribosomal protein S6 kinase and eIF4E-binding proteins. These authors also observed that liraglutide prevents pancreatic β-cell glucolipotoxicity via mTOR and that the actions of liraglutide are attenuated by AICAR, which is an AMPK activator, and rapamycin, which is an mTOR inhibitor (Miao et al., 2013).

The present study investigated rat primary hippocampal neurons and found that liraglutide promoted mTORC1 signaling activities under dexamethasone-induced toxic conditions. Liraglutide increased the phosphorylation of mTORC1 downstream effectors (p70S6K and 4E-BP-1) as well as the levels of BDNF and various synaptic proteins (PSD-95, Synapsin I, and GluA1) and enhanced synaptic density and neurite outgrowth. In addition to its effects on mTORC1 signaling activation, recent studies have shown that ketamine rapidly increases glutamate release and stimulates AMPA receptors, mTORC1 signaling, and synaptogenesis through the increased release of BDNF and enhanced AKT activation (Duman et al., 2012). Subsequently, released BDNF stimulates the syntheses of synaptic proteins, including PSD-95, synapsin I, and GluA1, in the prefrontal cortex of mice and contributes to the rapid antidepressant effects of ketamine (Duman et al., 2012; Dwyer and Duman, 2013; Abdallah et al., 2015). It has been proposed that PSD-95 plays an essential role in the maintenance and regulation of synaptic AMPA receptor function and AMPA receptor-dependent synaptic plasticity (Han and Kim, 2008). Increased levels of PSD-95 may be due to the increased number and size of dendritic spines and/or increases in the total number of synapses (Xu, 2011). Synapsin I is a modulator of neuronal development such that higher levels of synapsin I accelerate the development of synapses (Valtorta et al., 2011). GluA1 is a subunit of the AMPA receptor and is found in synapses where it enhances synaptic transmission (Santos et al., 2009). In the present study, liraglutide increased the expression of BDNF, PSD-95, synapsin I, and GluA1, whereas rapamycin and NBQX blocked the expression of each of these. In other studies, To date, no reports of studies investigating the activation of AMPA receptors on hippocampal neurons by liraglutide have been published. The present study is the first to report the effects of liraglutide on the activation of AMPA receptors in rat primary hippocampal neurons.

Chronic stress may cause depressive behavior by decreasing brain volume and reducing synaptic connections (Duman and Aghajanian, 2012). The activation of mTORC1 induces dendritic outgrowth but the induction of dendritic outgrowth is blocked by rapamycin (Park et al., 2014; Seo et al., 2016). Similarly, the present study demonstrated that liraglutide reversed decreases in neurite outgrowth in the dexamethasone-induced toxic condition and that the effects of liraglutide were blocked by rapamycin administration. Additionally, the increase in neurite outgrowth induced by liraglutide was blocked when NBQX and rapamycin were administered. Monnerie and Le Roux (2006) reported that kainate-induced dendritic outgrowth in mouse cortical neurons is blocked by NBQX; therefore, it can be postulated that the activations of mTORC1 signaling and AMPA receptors by liraglutide may promote neurite outgrowth. The effects of liraglutide on synaptic density were also observed in the present study. Liraglutide prevented reductions in synaptic density in the dexamethasone-induced toxic condition, but this effect was blocked by rapamycin and NBQX. These results suggest that liraglutide-induced changes in neurite outgrowth and synaptic density may occur via the activations of mTORC1 signaling and AMPA receptors.

In the present study, there are several limitations to this study that should be noted. First, it remains unclear whether the concentration of liraglutide used in the present study can be used in *in vivo* environments, particularly in humans. Therefore, it will be necessary to confirm the effects of liraglutide on mTORC1 signaling and AMPA receptors in further experiments using animal models. Second, AMPA receptor activity was assessed using the administration of NBQX. To measure the activity of the AMPA receptor more accurately, measurements of the AMPA-excitatory synaptic current should be obtained following the administration of liraglutide (Shinohara, 2012). Third, only 100 nM rapamycin and 10 nM NBQX doses were used in the study and future studies with additional concentrations of these drugs will be necessary.

**FIGURE 6:**
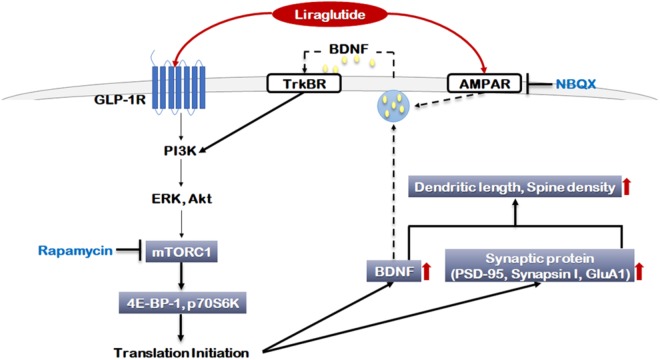
Signaling pathways regulated by liraglutide. The molecular pathways shown in gray boxes illustrate our novel observations. Akt: Protein kinase B (PKB), AMPAR: α-amino-3-hydroxy-5-methylisoxazole-4-propionic acid (AMPA) receptor, BDNF: brain-derived neurotrophic factor, ERK: extracellular signal-regulated kinase, GLP-1R: glucagon-like peptide 1 receptor, GluA1: AMPA receptor subunit GluR1, mTORC1: mammalian target of rapamycin complex 1, NBQX: 2,3-dioxo-6-nitro-1,2,3,4-tetrahydrobenzo[f]quinoxaline-7-sulfonamide, p70S6K: P70S6 kinase, PI3K: Phosphoinositide 3-kinase, PSD-95: Post Synaptic Density 95 protein, TrKBR: tropomyosin receptor kinase B receptor, 4E-BP-1: eukaryotic translation initiation factor 4E (eIF4E)-binding protein 1.

The present study was the first to investigate how liraglutide affects neuroplasticity via mTORC1 signaling and AMPA receptor activation. In this study, liraglutide activated mTORC1 signaling and AMPA receptors, increased the expression of BDNF and selected synaptic proteins, and enhanced neurite outgrowth and synaptic density under toxic conditions (Figure 6). Additionally, these liraglutide-induced changes were blocked by the mTORC1 inhibitor rapamycin and the AMPA receptor antagonist NBQX. These results indicate that liraglutide enacts neuroplastic regulation via mTORC1 signaling and AMPA receptor activation. Thus, it is possible that the activation of mTORC1 signaling and AMPA receptors may be a potential target for the modification of neural plasticity and the development of novel antidepressant drugs.

## Author Contributions

JL, SP, and RSM designed the study. MS and AC performed the experiments of this study. MS and SP wrote the protocol. SP and MS undertook the statistical analysis. RSM, RBM, YL, and J-HL contributed the methods and analysis tools. JL and SP wrote the first draft of the manuscript. All authors contributed to the approval of the final manuscript.

## Conflict of Interest Statement

The authors declare that the research was conducted in the absence of any commercial or financial relationships that could be construed as a potential conflict of interest.
