# Supplementary material for: Liraglutide Activates mTORC1 Signaling and AMPA Receptors in Rat Hippocampal Neurons Under Toxic Conditions
Source: Front Neurosci. 2018 Oct 23;12:756. doi: 10.3389/fnins.2018.00756 (PMC6205986; doi:10.3389/fnins.2018.00756)
Supplement: Supplementary file 1 [file Data_Sheet_1.docx]

Supplementary Material

Liraglutide Activates mTORC1 Signaling and AMPA Receptors in Rat Hippocampal Neurons under Toxic Conditions

Sung Woo Park^1,2,3^, Rodrigo B. Mansur^4,5^, Yena Lee^4^, Jae-Hon Lee^6^, Mi Kyoung Seo^1^, Ah Jeong Choi^1^, Roger S. McIntyre^4,5*^, and Jung Goo Lee^1,2,7*^

*** Correspondence:** Roger S. McIntyre: Roger.McIntyre@uhn.ca, Jung Goo Lee: iybihwc@naver.com


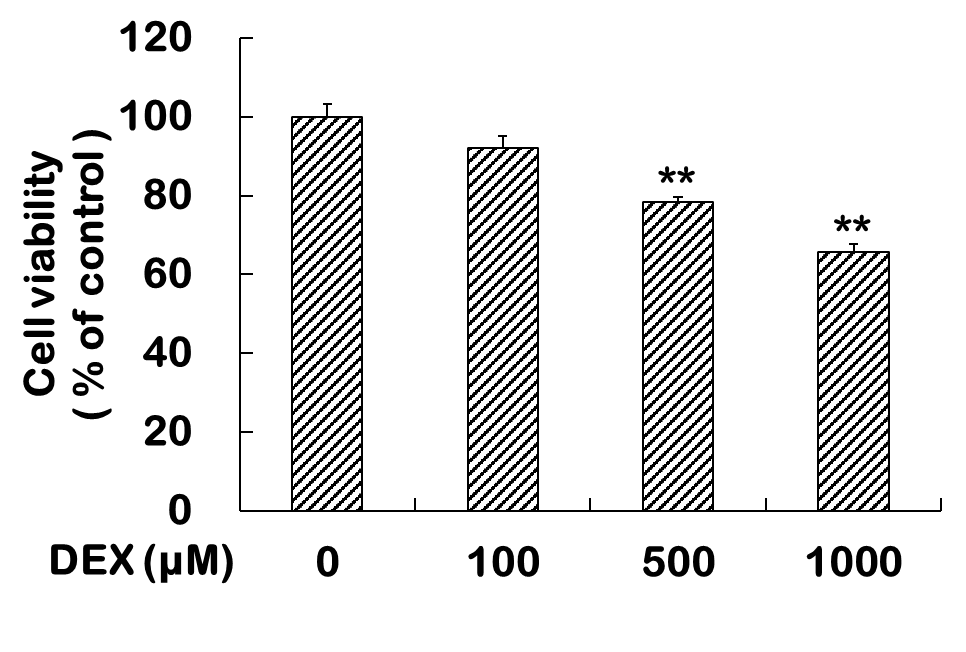


**Supplementary Figure 1. Dose-dependent cell death induced by dexamethasone in hippocampal cells.**

Cell were treated with either dexamethasone (100, 500, or 1000 μM) or DMSO (control, final concentration, 0.5%) for 4 days. An MTT assay was performed in two different wells per group of each of two independent cultures (total 10 wells); values (n = 10) are shown as mean ± standard error of the mean (SEM) expressed as the percentage of control cell values. ^**^*p* < 0.01 vs. control cells (DMSO treatment; one-way ANOVA followed by post hoc test).


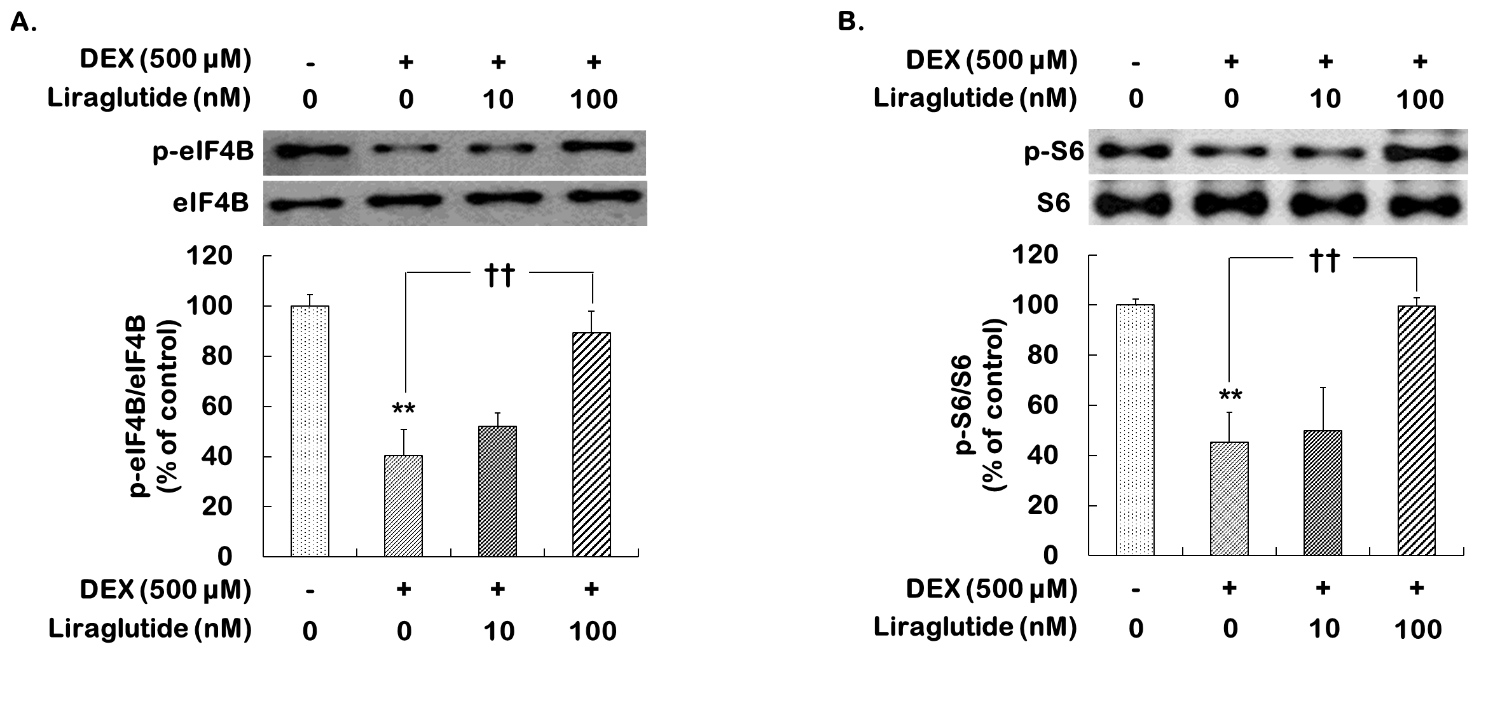


**Supplementary Figure 2. Effects of liraglutide on eIF4B and S6 phosphorylation in hippocampal cells treated with dexamethasone.**

Cells were treated with liraglutide (10 or 100 nM) or distilled water (control) for 4 days with (+; dexamethasone condition) or without (-; control condition) dexamethasone (DEX; 500 μM). In two different wells per group of each of two independent cultures (total 4 wells), cell lysates were analyzed by sodium dodecyl sulfate polyacrylamide gel electrophroesis (SDS-PAGE) and Western blotting with each of primary antibodies. The Western blot revealed the levels of phospho-Ser^422^-eIF4B (A) and phospho-Ser^240/244^-S6 (B). Representative images and quantitative analyses normalized to the total levels for each protein are shown; values (n = 4) are shown as the mean ± standard error of the mean (SEM) expressed as a percentage of the control cell values. ^**^*p* < 0.01 vs. control cells (-DEX, no liraglutide), ^††^*p* < 0.01 vs. dexamethasone-treated cells (+DEX, no liraglutide).

**Supplementary Table 1. Effects of liraglutide on total dendritic length and spine density in hippocampal cells under control condition.**

| Liraglutide (nM) | 0 | 10 | 100 |
| --- | --- | --- | --- |
| Total dendritic Outgrowth | 49.2±3.4 | 49.1±2.9 | 47.4±2.7 |
| Spine density | 1.4±0.1 | 1.5±0.2 | 1.4±0.1 |

Cells were treated with liraglutide (10 or 100 nM) or distilled water (control) for 5 days with without dexamethasone (control condition). Two independent cultures were performed. Cells were photographed and scored according to the methods described above. In total, 400 cells of group were analyzed for total dendritic length. In total, 40 dendritic segments per group were analyzed for spine density. All data (n = 400 for dendritic length, n = 40 for spine density) are expressed as mean ± SEM.
